# Histone deacetylase 3 is required for iNKT cell development

**DOI:** 10.1038/s41598-017-06102-5

**Published:** 2017-07-19

**Authors:** Puspa Thapa, Sinibaldo Romero Arocha, Ji Young Chung, Derek B. Sant’Angelo, Virginia Smith Shapiro

**Affiliations:** 1 Department of Immunology, Mayo Clinic, 200 First Street SW, Rochester, MN 55905 USA; 2 Department of Pediatrics, Rutgers Robert Wood Johnson Medical School and The Children’s Health Institute of New Jersey, 89 French Street, Room 4273, New Brunswick, NJ 08901 USA

## Abstract

NKT cells are a distinct subset that have developmental requirements that often differ from conventional T cells. Here, we show that NKT-specific deletion of Hdac3 results in a severe reduction in the number of iNKT cells, particularly of NKT1 cells. In addition, there is decreased cytokine production by Hdac3-deficient NKT2 and NKT17 cells. Hdac3-deficient iNKT cells have increased cell death that is not rescued by transgenic expression of Bcl-2 or Bcl-xL. Hdac3-deficient iNKT cells have less Cyto-ID staining and lower LC3A/B expression, indicative of reduced autophagy. Interestingly, Hdac3-deficient iNKT cells also have lower expression of the nutrient receptors GLUT1, CD71 and CD98, which would increase the need for autophagy when nutrients are limiting. Therefore, Hdac3 is required for iNKT cell development and differentiation.

## Introduction

Invariant Natural Killer T (iNKT) cells are an innate lineage of T cells characterized by the expression of an invariant Vα14-Jα18 TCR-α chain that pairs with limited TCRβ–chains Vβ7, or Vβ8 or Vβ2 in mice^1, 2^. The invariant TCR of iNKT cells recognizes glycolipids presented on MHC-like CD1d molecules^3^. They are a rare population in the thymus (~1%) and spleen (~1–2%), while 30% of lymphocytes in the liver are iNKT cells. iNKT cells produce copious amount of cytokines (including IFN-γ, IL-4, and IL-17) within minutes to hours of activation. iNKT cells are important for immunity against pathogens, autoimmune diseases and cancer^4^. The development of iNKT cells diverges from the conventional αβ T cells at the DP stage in the thymus^5, 6^. Upon positive selection into iNKT cell lineage, iNKT cells go through four developmental stages, Stage 0, Stage 1, Stage 2 and Stage 3. Newly selected Stage 0 iNKT cells express high levels of CD24^7^. Stage 1 iNKT cells undergo a burst of proliferation, regulated by the transcription factor c-Myc^8, 9^. At Stage 2, iNKT cells upregulate CD44. At Stage 3, iNKT cells express NK receptors, such as NK1.1 and require IL-15 for homeostasis^10, 11^. IL-15 signaling mediates survival of Stage 3 iNKT cells by regulating Bcl-xL expression. Transcription factors early growth response (Egr) 1 and Egr2 are also important for the expression of Bcl-2 in T cells^12, 13^. Loss of Egr2 in T cells leads to a block in iNKT cell development with increased cell death, supporting the importance of Bcl-2 in iNKT cell survival as well^13^.

Although the traditional linear developmental pathway was established to study iNKT cell development, it is now known that iNKT cells also differentiate into effector subsets in the thymus^14–16^. The functional effector subsets, NKT1, NKT2 and NKT17 are characterized by the transcription factors they express, Tbet, PLZF and ROR-γt, respectively, and the predominant production of IFN-γ, IL-4 and IL-17, respectively^17^. NKT2 cells develop in Stage 1 and Stage 2, NKT17 cell are found in Stage 2, whereas NKT1 cells are in Stage 3.

Autophagy is an evolutionarily conserved process critical for cell survival, differentiation and growth^18^. Autophagy is an intracellular degradation system where cytoplasmic proteins are delivered to the lysosome to be degraded and recycled. It is often triggered during nutrient deprivation, to serve as an alternate source of energy to sustain cellular function^19, 20^. During development and differentiation, iNKT cells undergo metabolic reprogramming to meet their changing energy demands. After positive selection, iNKT cells require autophagy for their transition from a proliferative state (Stage 1) to a more quiescent state at Stage 2 and Stage 3. During the proliferative burst at Stage 1, iNKT cells increase glycolysis^21^ while decreasing glucose uptake and increasing autophagy at Stage 2 and Stage 3. Loss of autophagy genes Atg5, Atg7, and Vps34 in T cells lead to a dramatic block in iNKT cell development but not conventional T cell development^21–23^. Requirement for autophagy in iNKT cells was cell intrinsic and not due to impaired CD1d-dependent-lipid antigen presentation to developing thymocytes, thus supporting the critical and unique role of autophagy in iNKT cell biology^21–23^.

Histone deacetylases (Hdacs) are histone-modifying enzymes that mediate removal of acetyl groups from proteins (histone and non-histone). Hdacs are essential for regulating expression of genes required for many biological processes. Hdac-mediated removal of acetyl groups from histones leads to epigenetic changes resulting in closed chromatin structure^24–26^. Hdac3 belongs to the Class I family of Hdacs and is ubiquitously expressed. Somatic deletion of Hdac3 is embryonically lethal^27^. Hdac3 is required for hematopoietic stem cell (HSC) survival^28^. Hdac3 is also required for positive selection of conventional αβ T cells and iNKT cells in the thymus^29–31^. Peripheral recent thymic emigrants (RTEs) CD4 and CD8 T cells also depend on Hdac3 for their maturation^32^.

Although the requirement of autophagy during iNKT cell development has been described, the regulation of autophagy during iNKT cell development is not fully understood. In this study we show a potential role for Hdac3 in regulating autophagy for proper development of iNKT cells. Using PLZF-cre Hdac3 cKO mice, we demonstrate loss of Hdac3 leads to a decreased autophagy and a severe defect in iNKT cell numbers, particularly of NKT1 effector cells. While NKT2 and NKT17 cells develop, they have decreased ability to produce IL-4 and IL-17, respectively. Moreover, in the absence of Hdac3, there is increased iNKT cell death that cannot be rescued by transgenic expression of Bcl-2 or Bcl-xL. Therefore, the transcriptional regulator Hdac3 plays a critical role in regulation of iNKT cell survival and effector function during development.

## Results

### Hdac3 is required for iNKT cell development

**Figure 1:**
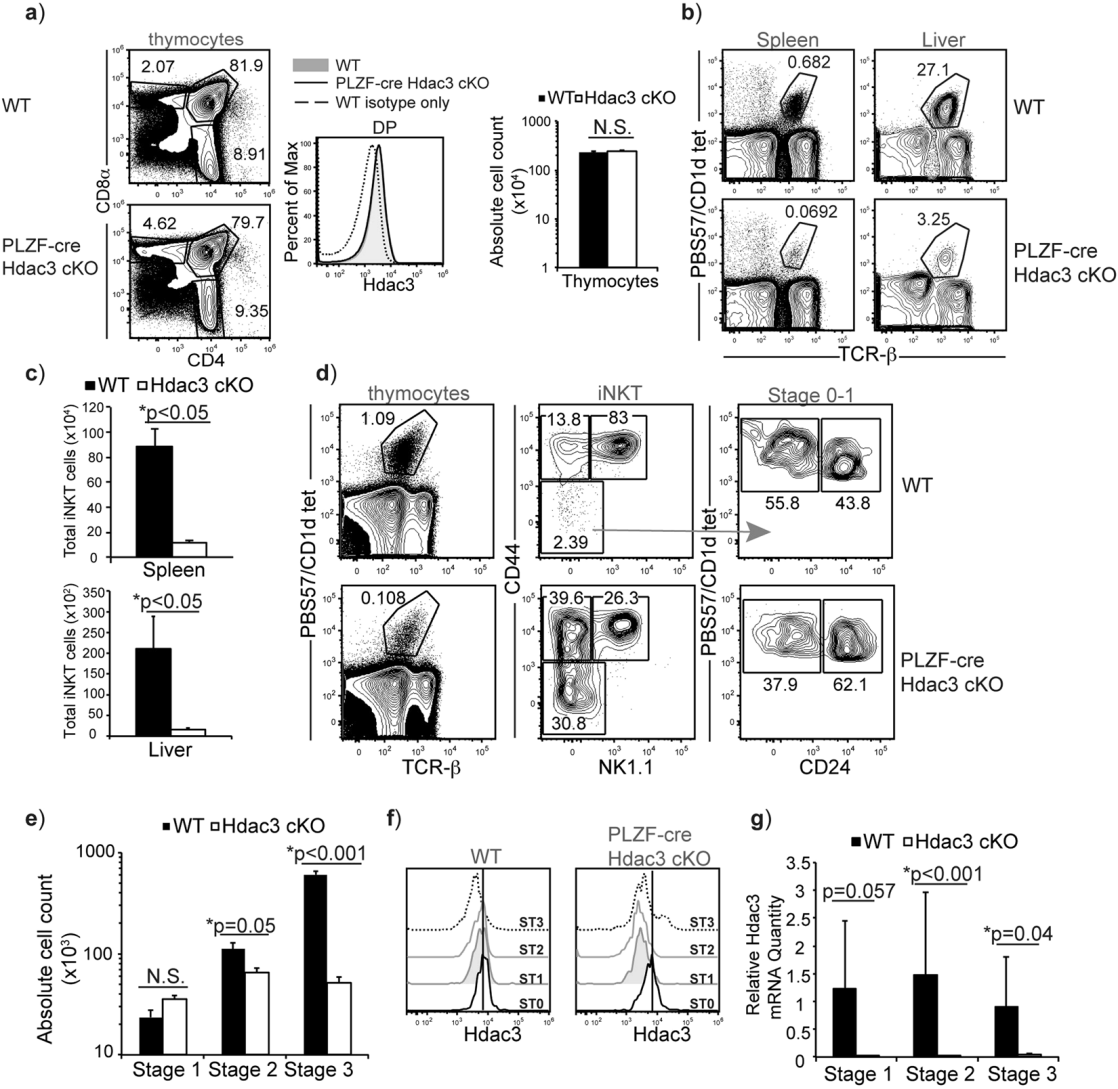
Hdac3 is required for iNKT cell development. (**a**) CD4/CD8 profile in thymocytes from WT and PLZF-cre Hdac3 cKO mice. Hdac3 expression in DP thymocytes from WT and PLZF-cre Hdac3 cKO mice as well as isotype control in WT mice is shown. Data is representative of at least 5 mice/genotype from 4 independent experiments. Absolute cell count of thymocytes from WT and PLZF-cre Hdac3 cKO mice. Data is calculated from 21 WT and 12 PLZF-cre Hdac3 cKO mice. (**b**) Representative frequencies of iNKT cells in spleen and liver. Data is representative of at least 5 mice/genotype. (**c**) Quantification of absolute cell number of iNKT cells (CD1d:PBS57 tetramer^+^ TCR-β^med^) from spleen and liver of WT and PLZF-cre Hdac3 cKO mice. Data is calculated from 7 mice/genotype (spleen) and 5 mice/genotype (liver) from 3 independent experiments. (**d**) FACS analysis of thymic iNKT cells in developmental stages was performed using CD24, CD44 and NK1.1: Stage 0 (CD24^+^ CD44^−^ NK1.1^−^), Stage 1 (CD24^−^ CD44^lo^ NK1.1^−^), Stage 2 (CD24^−^ CD44^hi^ NK1.1^−^), and Stage 3 (CD24^−^ CD44^+^ NK1.1^+^). Data is representative of at least 12 mice per genotype. (**e**) Absolute cell count of thymic iNKT cells at Stage 1, Stage 2 and Stage 3. Average is calculated from 21 WT and 12 PLZF-cre Hdac3 cKO mice from 6–10 weeks of age. (**f**) Overlay of expression of Hdac3 in Stage 0–3 iNKT cells from WT and PLZF-cre Hdac3 cKO mice. Data is representative of at least 6 mice per genotype from 5 independent experiments. Stage 0: black line, Stage 1, grey filled, Stage 2: grey line, Stage 3: dashed line. Black vertical line represents peak of Hdac3 expression in WT and PLZF-cre Hdac3 cKO Stage 0 iNKT cells. (**g**) Relative expression of *Hdac3* mRNA in sorted iNKT cells in WT and PLZF-cre Hdac3 cKO using q-PCR. Data was normalized to expression of *Hdac3* in WT Stage 3 iNKT cells (=1). Data is calculated from 3 WT and 5 PLZF-cre Hdac3 cKO mice from 3 independent sorts. Statistical analysis was performed using a Student *t* test. Means ± SEM.

Previously, using CD4-cre Hdac3 cKO mice, we found that Hdac3 is required for positive selection of DP thymocytes into the iNKT cell lineage^31^. To determine whether Hdac3 has additional roles in later iNKT cell development and differentiation, we generated PLZF-cre Hdac3 cKO mice where deletion in thymocytes initiates after selection into the iNKT cell lineage. Using lineage tracing, it was previously determined that PLZF-cre is transiently expressed in a hematopoietic stem cell progenitor, leading to labeling of approximately 30% of the HSC pool^33, 34^. However, as Hdac3 is required for HSC survival^28^, any Hdac3-deficient HSC will not persist and contribute to hematopoiesis in an adult mouse. Previously, it was shown that (using lck-cre or CD2-icre) Hdac3 is absolutely required for positive selection of DP thymocytes into CD4 and CD8 SP thymocytes^29, 30^. If Hdac3 was deleted in early T cell progenitors in PLZF-cre Hdac3 cKO mice, then a block in conventional T cell development would have been observed^29, 30^. However, conventional CD4 and CD8 T cell development is unaltered in PLZF-cre Hdac3 cKO mice (Fig. 1a). Additionally, the absolute number of thymocytes and the expression of Hdac3 in DP thymocytes is equivalent in WT and PLZF-cre Hdac3 cKO mice (Fig. 1a). Thus, there is no deletion of Hdac3 in DP thymocytes, allowing us to use PLZF-cre to study Hdac3’s function specifically in the iNKT cell lineage. Using flow cytometry, we examined iNKT cell development in PLZF-cre Hdac3 cKO mice. There was an approximately 10-fold reduction in splenic and liver iNKT cells in PLZF-cre Hdac3 cKO mice (Fig. 1b and c). There was a similar 10-fold decrease in thymic iNKT cells (Fig. 1d). In addition, the majority of iNKT cells in the thymus were skewed towards earlier stages (Stage 0–2) in development. The absolute cell count revealed a significant ~2 fold reduction (p < 0.025) in the absolute cell number of iNKT cells at Stage 2, and dramatic ~11.8 fold reduction (p < 0.001) at Stage 3 (Fig. 1e). Protein expression of Hdac3 was examined in Stages 0–3 iNKT cells of WT and PLZF-cre Hdac3 cKO using flow cytometry. Expression of Hdac3 was similar in Stage 0 iNKT cells of WT and PLZF-cre Hdac3 cKO mice. Reduction of Hdac3 protein level was observed starting in Stage 1 in the PLZF-cre Hdac3 cKO mice (Fig. 1f). Thus, as Stage 0 iNKT cells in PLZF-cre Hdac3 cKO mice have normal Hdac3 protein expression, they were not analyzed further. Quantification of relative *Hdac3* mRNA expression using q-PCR revealed that *Hdac3* was efficiently deleted in iNKT cells in the PLZF-cre Hdac3 cKO mice, confirming the deletion of Hdac3 from Stage 1 onwards (Fig. 1g). The expression of CD1d was also unaltered in DP thymocytes or iNKT cells from PLZF-cre Hdac3 cKO mice, demonstrating that the defect is not due to decreased CD1d expression (Supplemental Fig. 1). Therefore, Hdac3 is required for iNKT cell development and loss of Hdac3 has a severe defect particularly in Stage 3 iNKT cell numbers.

### Deletion of Hdac3 leads to a loss in Tbet expressing NKT1 cells

**Figure 2:**
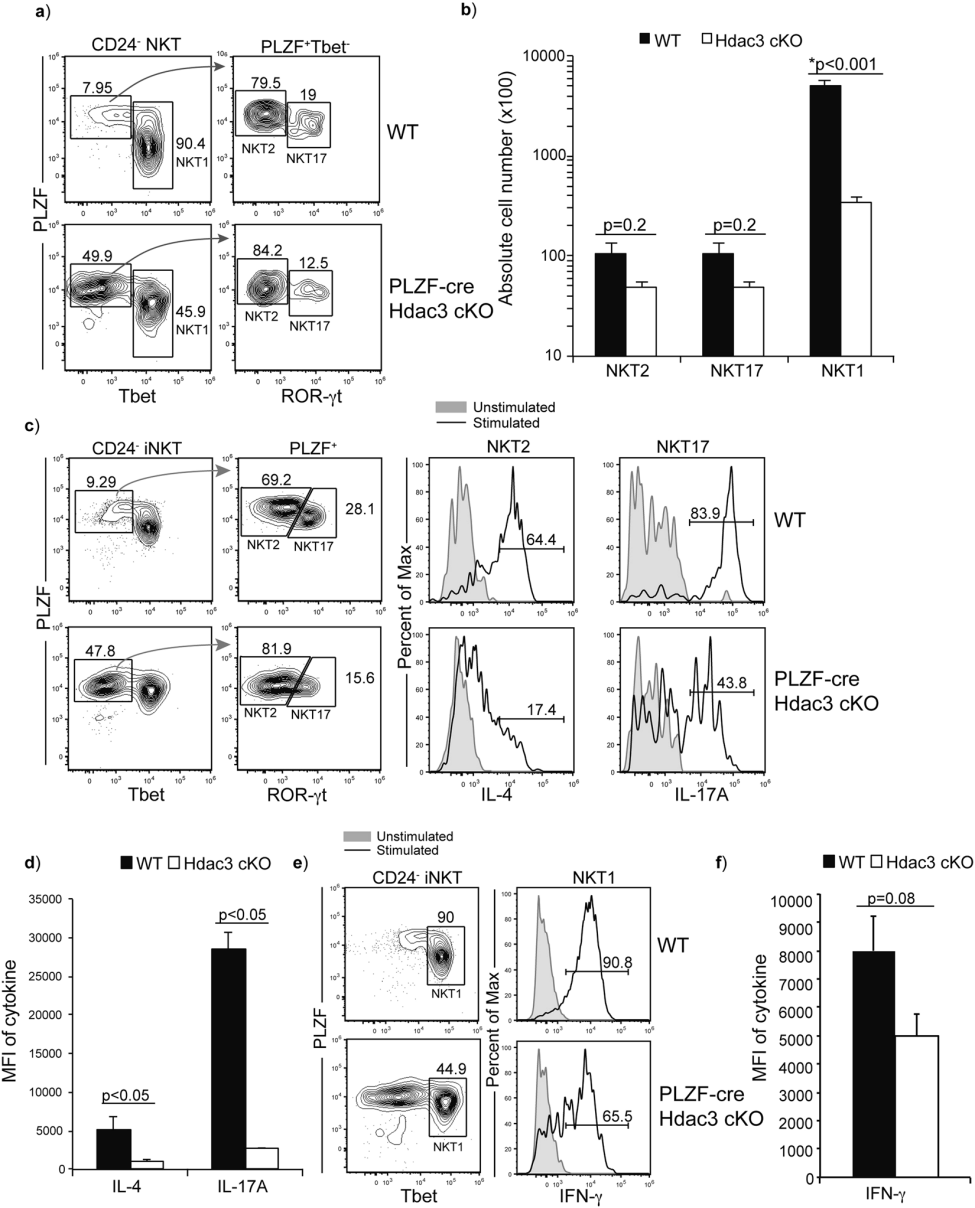
Hdac3-deficient iNKT cells have a defect in the differentiation of Tbet expressing NKT1. (**a**) Examination of thymic NKT subsets (NKT1, NKT2 and NKT17) as characterized by the expression of Tbet, PLZF and ROR-γt. NKT1 (Tbet^+^ PLZF^med^), NKT2 (PLZF^hi^ ROR-γt^−^ Tbet^−^) and NKT17 (ROR-γt^+^ PLZF^med^ Tbet^−^) in WT and PLZF-cre Hdac3 cKO mice. Data is representative of at least 8 mice per genotype. (**b**) Absolute cell count for NKT2, NKT17 and NKT1 cells from WT (black bar) and PLZF-cre Hdac3 cKO (white bar) mice. Data is calculated from 14 WT and 8 PLZF-cre Hdac3 cKO mice. Statistical analysis was performed using a Student *t* test. Means ± SEM. (**c**) Production of IL-4 and IL-17A by stimulated (black line) and unstimulated (grey filled) NKT2 and NKT17 cells in the WT and PLZF-cre Hdac3 cKO after 6 hr stimulation with PMA/ionomycin. NKT subsets were defined as described in (**a**). Data is representative of at least 5 mice per genotype from 3 independent experiments. (**d**) Quantification of average MFI of IL-4 and IL-17A production by NKT2 and NKT17, respectively from WT (black bar) and PLZF-cre Hdac3 cKO (white bar). Data is calculated from 5 WT and 5 PLZF-cre Hdac3 cKO mice. Statistical analysis was performed using a Student *t* test. Means ± SEM. (**e**) Production of IFN-γ by stimulated (black line) and unstimulated (grey filled) NKT1 cells in the WT and PLZF-cre Hdac3 cKO after 6hr stimulation with PMA/ionomycin. NKT subsets were defined as described in (**a**). Data is representative of 5 mice per group. (**f**) Quantification of average MFI of IFN-γ production by NKT1 from WT (black bar) and PLZF-cre Hdac3 cKO (white bar) mice. Data is calculated from 5 WT and 5 PLZF-cre Hdac3 cKO mice. Statistical analysis was performed using a Student *t* test. Means ± SEM.

Although iNKT cell development was originally described as a linear progression, it is now established that iNKT cells differentiate into effector subsets, NKT1, NKT2 and NKT17, in the thymus^14^. The functional subsets, NKT1, NKT2 and NKT17 are characterized by the expression of transcription factors Tbet, PLZF and ROR-γt and the predominant production of cytokines IFN-γ, IL-4 and IL-17, respectively^14, 16, 17^. Aligning the functional subsets with the traditional staging, NKT1 cells are restricted to NK1.1 expressing Stage 3, while NKT2 cells are found in both Stage 1 and Stage 2, and NKT17 cells are found in Stage 2. Utilizing the expression of PLZF, Tbet and ROR-γt to identify all three subsets, we used Tbet^+^ PLZF^low^ to identify NKT1 cells, and PLZF^+^ Tbet^−^ iNKT cells were further delineated based on PLZF and ROR-γt expression to identify NKT2 and NKT17 cells. NKT2 cells are PLZF^hi^ ROR-γt^−^ and NKT17 cells are ROR-γt^+^ PLZF^med^ (Fig. 2a). In PLZF-cre Hdac3 cKO mice, we observed a severe reduction in proportion of NKT1 cells, which is consistent with the reduction in NK1.1^+^ Stage 3 iNKT cells (Fig. 2a). The proportion of NKT17 cells and NKT2 cells in Tbet^−^ iNKT cell population was similar in PLZF-cre Hdac3 cKO mice and WT mice, although there is a ~2 fold reduction in absolute cell number which did not reach statistical significance (Fig. 2a,b). Thus, in the absence of Hdac3, there are few NKT1 cells present. To determine if Hdac3 also regulates iNKT cell function, we examined production of cytokines (IFN-γ, IL-4 and IL-17A) by the NKT subsets (NKT1, NKT2 and NKT17, respectively) in WT and PLZF-cre Hdac3 cKO mice. Hdac3-deficient NKT2 and NKT17 cells had significantly reduced ability to produce IL-4 and IL-17A upon stimulation for 6 hours with PMA/ionomycin, as measured by MFI in each population (Fig. 2c,d). However, production of IFN-γ after stimulation was not statistically different between NKT1 cells from WT and PLZF-cre Hdac3 cKO mice (Fig. 2e,f). Within each stimulated population, Hdac3-deficient NKT1, NKT2 and NKT17 cells had lower proportion of cells that produced cytokines upon stimulation as compared to WT (Supplemental Fig. 1) This difference in cytokine production is not due to decreased viability upon stimulation (Supplemental Fig. 1). Overall, these results show that loss of Hdac3 leads to generation of fewer NKT1 cells, but not NKT2 and NKT17 cells and less IL-4 and IL-17A is produced by NKT2 and NKT17, respectively, upon stimulation.

### Hdac3-deficient iNKT cells go through normal proliferative burst at Stage1, but exhibit increased cell death

**Figure 3:**
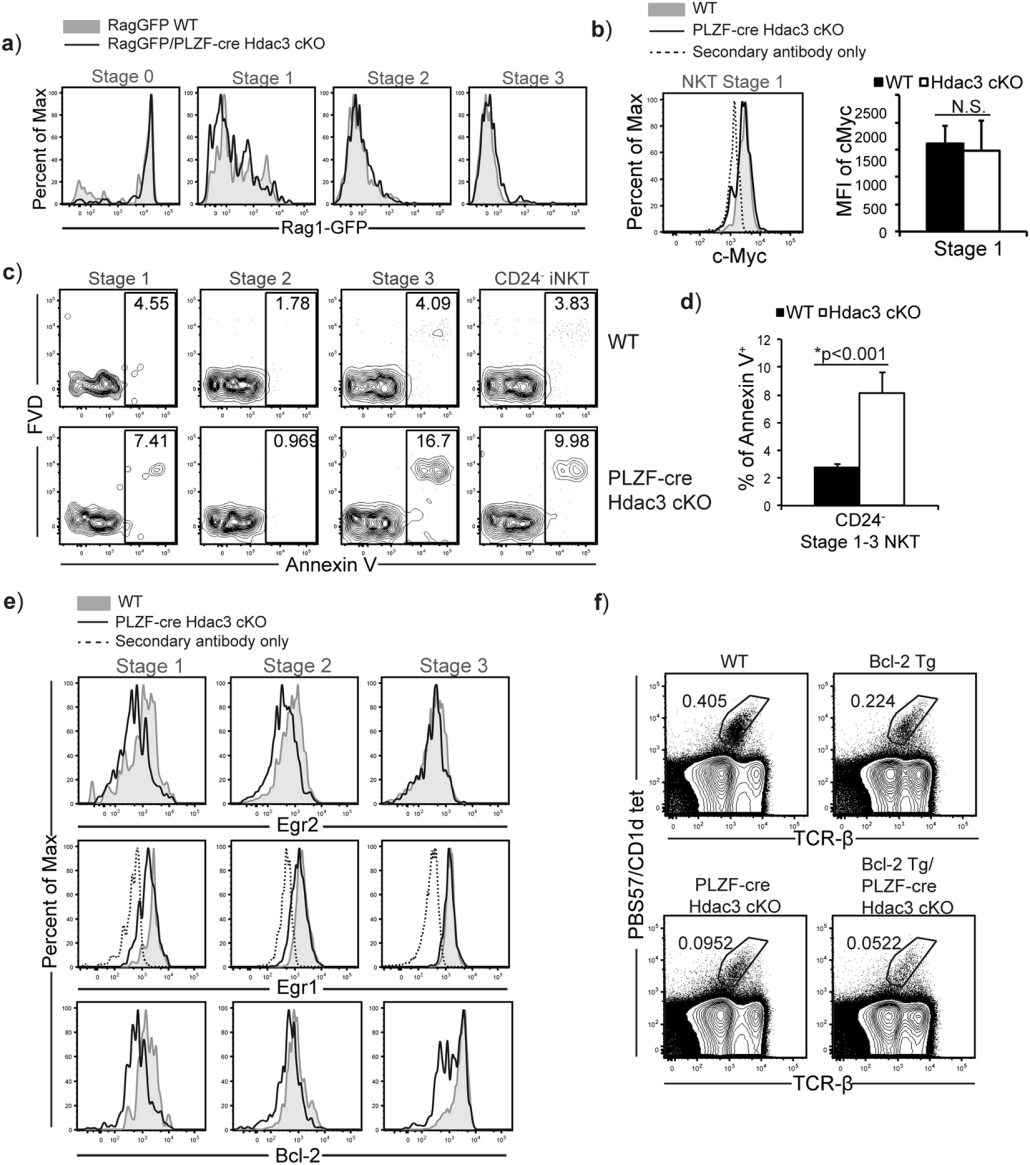
Normal proliferation but increased cell death in Hdac3-deficient iNKT cells. (**a**) FACS analysis of GFP expression in iNKT cells at their developmental stages (Stage 0–3) in Rag1-GFP (WT) (grey filled) and Rag1-GFP/PLZF-cre Hdac3 cKO (black line). Data is representative of at least 4 mice/genotype from 4 independent experiments. (**b**) Intracellular expression of c-Myc in Stage 1 iNKT cells of WT (grey filled) and PLZF-cre Hdac3 cKO (black line); secondary antibody only control is shown with dashed line. Data is representative of at least 3 mice/genotype from at least 3 independent experiments. Quantification of average MFI of c-Myc from Stage 1 iNKT cells of WT (black bar) and PLZF-cre Hdac3 cKO (white bar) mice. Data is calculated from 5 WT and 3 PLZF-cre Hdac3 cKO mice. Statistical analysis was performed using a Student *t* test. Means ± SEM. (**c**) Representative frequency of apoptotic Stage 1 (CD24^−^ CD44^lo^ NK1.1^−^), Stage 2 (CD24^−^ CD44^hi^ NK1.1^−^), and Stage 3 (CD24^−^ NK1.1^+^). iNKT cells in WT (top) and PLZF-cre Hdac3 cKO (bottom) mice using Annexin V^+^ and Fixable Viability Dye^+^ (FVD). Data is representative of at least 9 mice per genotype from 7 independent experiments. (**d**) Quantification of frequency of apoptotic Annexin V^+^ CD24^−^ iNKT cells. Average was calculated from 15 WT (black bars) and 9 PLZF-cre Hdac3 cKO (white bars) mice from at least 7 independent experiments. Statistical analysis was done using a Student *t* test. Means ± SEM. (**e**) Expression of Egr1, Egr2 and their target Bcl-2 in Stage 1–3 iNKT cells of WT (grey filled), PLZF-cre Hdac3 cKO (black line) mice. Secondary antibody (fluorescent labeled) only control (dashed) is shown for Egr1 where unlabeled primary antibody against Egr1 was used. Egr2 and Bcl-2 antibodies were directly conjugated to fluorophores. Data is representative of at least 4 mice/genotype from 3 independent experiments. (**f**) Proportion of iNKT cells in WT, PLZF-cre Hdac3 cKO, Bcl-2 Tg and Bcl-2 Tg/ PLZF-cre Hdac3 cKO mice. Data is representative of at least 3 mice/genotype from 3 independent experiments. Staining and gating for each iNKT cell stages was performed as described in Fig. 1.

At Stage 1, iNKT cells undergo a burst of proliferation; one explanation for the decreased iNKT cell number in the absence of Hdac3 may be due to a defect in proliferation. To investigate the rate of proliferation, we utilized Rag1-GFP reporter mice^35^. Here, GFP is knocked into one allele of *Rag1* locus. The transcription of *Rag1* is turned off at the DP thymocyte stage after successful rearrangement of the TCR α chain. Although transcription of *Rag1* (and *GFP*) stops at the DP stage, GFP protein is stable with a long half-life^36^. In the periphery, Rag1-GFP reporter expression is used to identify recent thymic emigrants (RTE), as conventional T cells do not proliferate after the DP stage. However, iNKT cells proliferate after selection into the iNKT cell lineage at Stage 1. Therefore, the expression of GFP will be diluted as they undergo proliferation at Stage 1, and the dilution of GFP in iNKT cells can function similar to an ‘*in vivo* CFSE labeling’ in iNKT cells to measure rate of proliferation^35^. We generated Rag1-GFP/PLZF-cre Hdac3 cKO mice and in WT (Rag1-GFP) mice, Stage 0 iNKT cells expressed high levels of GFP. As iNKT cells progress through Stage 1 and Stage 2, the expression of GFP is diluted by proliferation. By Stage 3, all iNKT cells have lost Rag1-GFP reporter expression. The expression of GFP in Rag1-GFP/PLZF-cre Hdac3 cKO mice in all stages of iNKT cell development was similar to Rag1-GFP (WT) mice, indicating that decreased iNKT cell number is not due to a defect in proliferation (Fig. 3a). Expression of the transcription factor c-Myc^8, 9^, which is required for proliferation of iNKT cells, was also normal in Stage 1 iNKT cells from PLZF-cre Hdac3 cKO mice (Fig. 3b). The expression of proliferative capacity marker Ki-67 was not significantly different at each stage of iNKT cell development PLZF-cre Hdac3 cKO mice as compared WT mice (Supplemental Fig. 2). Thus, the cause for the lower number of iNKT cells in PLZF-cre Hdac3 cKO mice is not due to decreased proliferation. We next examined whether the lower number of iNKT cells in the absence of Hdac3 could be due to increased cell death. Using a fixable viability dye (FVD) and Annexin V, we found increased Annexin V^+^ apoptotic iNKT cells in the PLZF-cre Hdac3 cKO mice as compared to WT (Fig. 3c). Quantification of the frequency of apoptotic CD24^−^ iNKT cells in PLZF-cre Hdac3 cKO mice revealed ~3-fold (p < 0.005) increase as compared to WT mice (Fig. 3d). Therefore, lack of Hdac3 led to decreased viability in iNKT cells. In developing T cells, the transcription factors Egr1 and Egr2 are required for the expression of pro-survival gene Bcl-2^12^. Remarkably, in Stage 1 and Stage 2 Hdac3-deficient iNKT cells, we observed decreased expression of Egr1 and Egr2 as compared to WT. Furthermore, the expression of their target gene Bcl-2 was also reduced in Hdac3-deficient iNKT cells (Fig. 3e). The MFI of Egr1 and Egr2 expression was significantly reduced in Hdac3-deficient iNKT cells compared to WT, while the MFI of Bcl-2 was also reduced although it did not reach significance (Supplemental Fig. 2). To address if decreased expression of pro-survival gene Bcl-2 was the cause for the increased apoptosis observed in the PLZF-cre Hdac3 cKO mice, we generated Bcl-2 transgenic/PLZF-cre Hdac3 cKO mice. However the deficiency in iNKT cell numbers was not rescued by the overexpression of Bcl-2 (Fig. 3f). Thus, loss of Hdac3 leads to decreased viability that is not due to decreased Bcl-2 expression.

### Block in development is not due to dysregulated iNKT cell homeostasis

**Figure 4:**
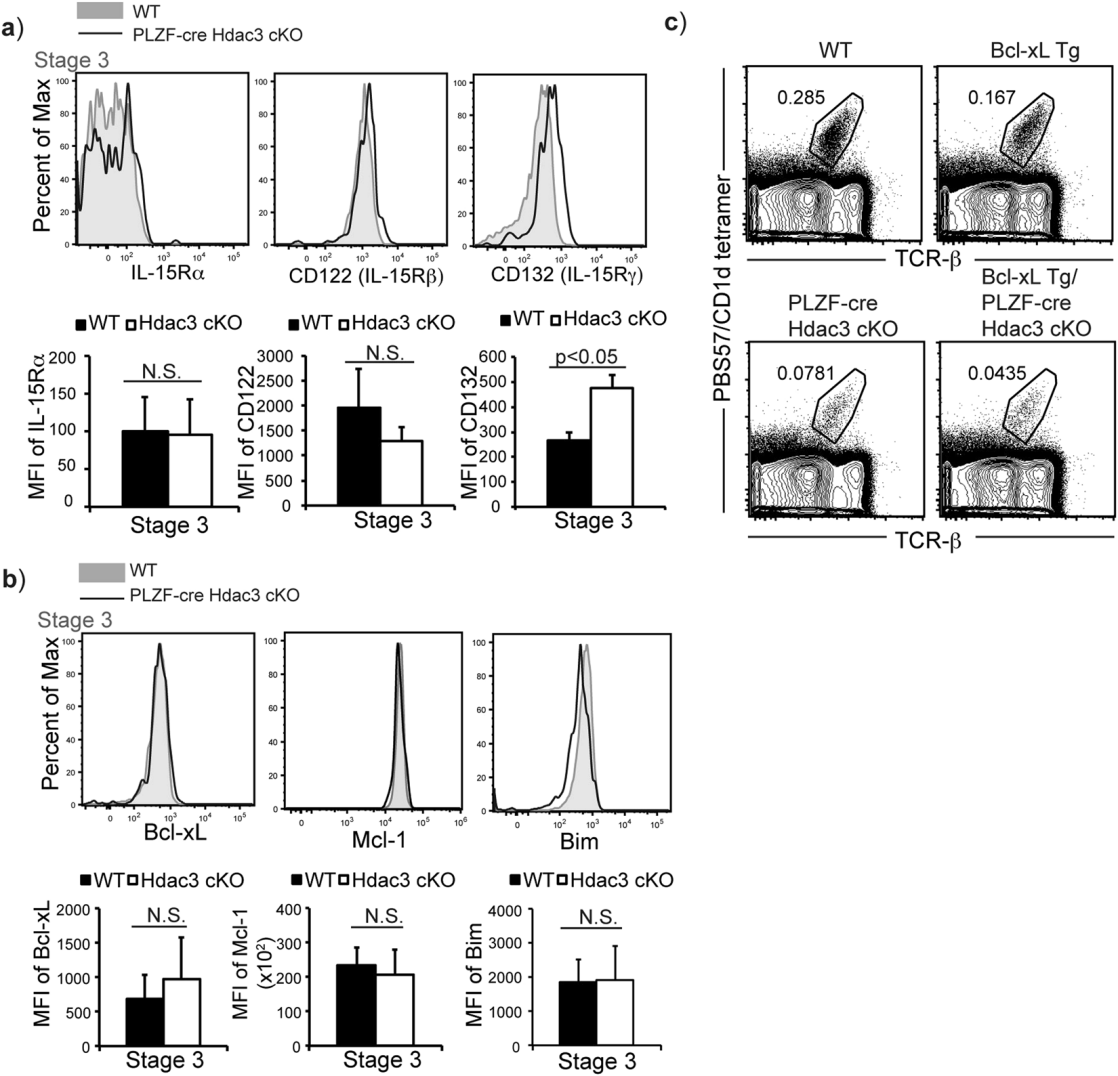
Block in iNKT cell development in PLZF-cre Hdac3 cKO cannot be rescued by Bcl-xL transgene expression. (**a**) Surface expression of IL-15 receptors: IL-15Rα, IL15Rβ (CD122) and IL-15Rγ (CD132) in Stage 3 iNKT cells of WT (grey filled) and PLZF-cre Hdac3 cKO (black line). Data is representative of at least 4 mice/genotype from 3 independent experiments. Quantification of average MFI of IL-15Rα, IL15Rβ (CD122) and IL-15Rγ (CD132) in Stage 3 iNKT cells of WT (black bar) and PLZF-cre Hdac3 cKO (white bar) mice. Data is calculated from 4 WT and 4 PLZF-cre Hdac3 cKO mice. Statistical analysis was performed using a Student *t* test. Means ± SEM. (**b**) Intracellular expression of anti-apoptotic molecules: Bcl-xL, Mcl-1, as well as pro-apoptotic molecule, Bim in Stage 3 iNKT cells of WT (grey filled) and PLZF-cre Hdac3 cKO (black line). Data is representative of at least 3 mice/genotype from 2 independent experiments. Quantification of average MFI of Bcl-xL, Mcl-1 and Bim in Stage 3 iNKT cells of WT (black bar) and PLZF-cre Hdac3 cKO (white bar) mice. Data is calculated from 4 WT and 4 PLZF-cre Hdac3 cKO mice. Statistical analysis was performed using a Student *t* test. Means ± SEM. (**c**) Representative frequency of iNKT cells in WT, Bcl-xL Tg, PLZF-cre Hdac3 cKO and Bcl-xL Tg/ PLZF-cre Hdac3 cKO mice. Data is representative of at least 3 mice/genotype from 2 independent experiments. Staining and gating for each iNKT cell stages was performed as described in Fig. 1.

Stage 3 iNKT cells require IL-15 for their homeostasis and survival^10, 11^. Deletion of IL-15 in T cells lead to severe reduction in Stage 3 iNKT cells but not earlier stages of iNKT cell development. The IL-15R is comprised of 3 subunits, which are IL-15Rα, CD122 (IL-15Rβ) and CD132 (IL-15Rγ, common γc). There was no difference in expression of IL-15Rα and CD122, while expression of CD132 was increased in Stage 3 iNKT ells from PLZF-cre Hdac3 cKO mice (Fig. 4a). IL-15 signaling regulates the expression of pro-survival gene Bcl-xL in iNKT cells. In addition, Hdac3-deficient iNKT cells did not have significant changes in the expression of Bcl-xL, pro-survival molecule Mcl-1, or pro-apoptotic molecule Bim (Fig. 4b). Previous reports demonstrated that the dysregulated homeostasis of iNKT cells in the IL-15 KO mice was rescued by the over-expression of Bcl-xL^10^. However, transgenic expression of Bcl-xL did not rescue the defect in iNKT cell numbers in the Bcl-xL Tg/PLZF-cre Hdac3 cKO mice (Fig. 4c). Hence, the severe decrease in Stage 3 iNKT cell numbers in the absence of Hdac3 is not due to dysregulated iNKT cell homeostasis involving IL-15 receptor or Bcl-xL expression.

### Hdac3-deficient iNKT cells exhibit reduced autophagy

**Figure 5:**
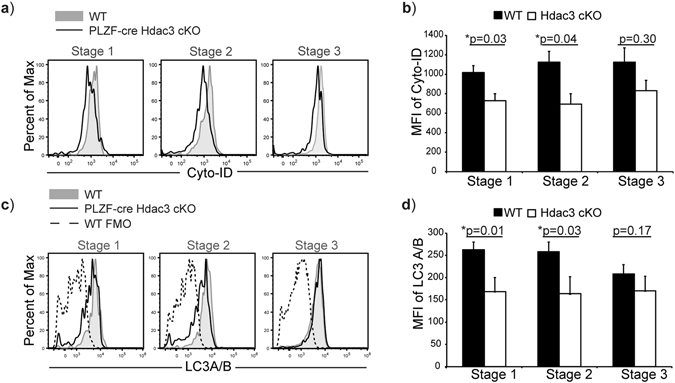
Hdac3-deficient iNKT cells exhibit reduced autophagy. (**a**) Overlay of expression of Cyto-ID stain in Stage 1–3 iNKT cells in WT (grey filled) and PLZF-cre Hdac3 cKO mice (black line). Data is representative of at least 5 WT and 4 PLZF-cre Hdac3 cKO mice from 3 independent experiments. (**b**) Quantification of average MFI of Cyto-ID intake by Stage 1–3 iNKT cells in WT (black bar) and PLZF-cre Hdac3 cKO (white bar) mice. Data is calculated from 5 WT and 4 PLZF-cre Hdac3 cKO mice from 3 independent experiments. Statistical analysis was done using student’s *t* test. Means ± SEM. (**c**) Examination of total LC3A/B in Stage 1–3 iNKT cells from WT (grey filled) and PLZF-cre Hdac3 cKO (black line) mice. For the LC3A/B stain, WT fluorescence minus one (FMO) is shown as a control. Data is representative of at least 5 WT and 4 PLZF-cre Hdac3 cKO mice from 3 independent experiments. (**d**) Quantification of average MFI of LC3A/B expression by Stage 1–3 iNKT cells in WT (black bars) and PLZF-cre Hdac3 cKO (white bars) mice. Data is calculated from 5 WT and 4 PLZF-cre Hdac3 cKO mice from 3 independent experiments. Statistical analysis was done using student’s *t* test. Means ± SEM.

Autophagy is highly regulated during iNKT cell development. Deletion of Vps34, Atg5 or Atg7, which are each required for autophagy, leads to a block in iNKT cell development with a dramatic decrease in iNKT cell frequencies and cell numbers^21–23^. Atg5-deficient iNKT cells have significant reduction in cell number at Stage 2 and Stage 3, while Atg7-deficient iNKT cells are arrested at Stage 2 with significant reduction in NK1.1^+^ iNKT cells^21, 23^. In the absence of Vps34, a severe decrease in the proportion of NK1.1^+^ iNKT cells was observed as well^22^. Atg5-deficient iNKT cells exhibited increased cell death coupled with cell cycle arrest and increased mitochondrial stress. Atg5-deficient iNKT cells also had increased proportions of NKT2 and NKT17 with severe reduction of NKT1 cells, correlating to loss of NK1.1^+^ iNKT cells. Furthermore, loss of Atg5 in iNKT cells also lead to reduced production of IL-4 and IFN-γ^23^. Whereas, Atg7-deficient iNKT cells exhibited normal proliferation but had increased apoptosis. Loss of Atg7 also leads to decreased expression of Egr2 and its target Bcl-2^21^. Atg7-deficient iNKT cells also had a defect in differentiating to NKT1 subsets, which correlates, to decreased NK1.1^+^ iNKT cell numbers. In CD4-cre Atg7 cKO mice, there is reduced production of IL-17 and IL-4 by NKT cells^21^. Loss of Hdac3 in iNKT cells mirrors defects exhibited by Atg5 and Atg7-deficient NKT cells; thus, we hypothesized that the cause for the defects in Hdac3-deficient iNKT cells could be due to a defect in autophagy. We examined autophagy in iNKT cells by measuring Cyto-ID staining and LC3A/B expression in WT and PLZF-cre Hdac3 cKO mice. Cyto-ID staining measures autophagic flux in live cells by selectively labeling autophagic vacuoles that accumulate during autophagy^37, 38^, and thus is used to quantify active autophagy. LC3A/B (Atg8) is a subunit of the autophagy complex that is critical for the formation and transportation of autophagic vesicles to endosomal vacuoles during autophagy^39^; thus, total levels of LC3A/B can be used as measurement of autophagic function. The levels of both Cyto-ID staining and LC3A/B expression was reduced in Hdac3-deficient iNKT cells compared to WT iNKT cells (Fig. 5a,c). Quantification of the MFI of Cyto-ID staining and LC3A/B in Stage 1–3 iNKT cells revealed a statistically significant reduction in Cyto-ID staining and LC3A/B expression in Stage 1 and Stage 2 iNKT cells from PLZF-cre Hdac3 cKO mice compared to WT mice (Fig. 5b,d). We examined expression of two genes required for autophagy, Atg7 and p62. The expression of Atg7 was not altered in Hdac3-deficient iNKT cells compared to WT (Supplemental Fig. 3). The expression of p62, a receptor important for cargo destined to be degraded by autophagy, was also similar in Hdac3-deficient Stage 1–3 iNKT cells compared to the WT Stage 1–3 iNKT cells (Supplemental Fig. 3). Cell size across Stages 1–3 was similar between WT and Hdac3-deficient iNKT cells, indicating that decreased Cyto-ID staining or LC3A/B expression is not due to cell size differences (Supplemental Fig. 3). Taken together, loss of Hdac3 leads to reduction in autophagy, which is important for iNKT cell development and survival, although independently of either ATG7 and p62 expression.

### Hdac3-deficient iNKT cells exhibit reduced expression of nutrient receptors, GLUT1, CD71 and CD98

**Figure 6:**
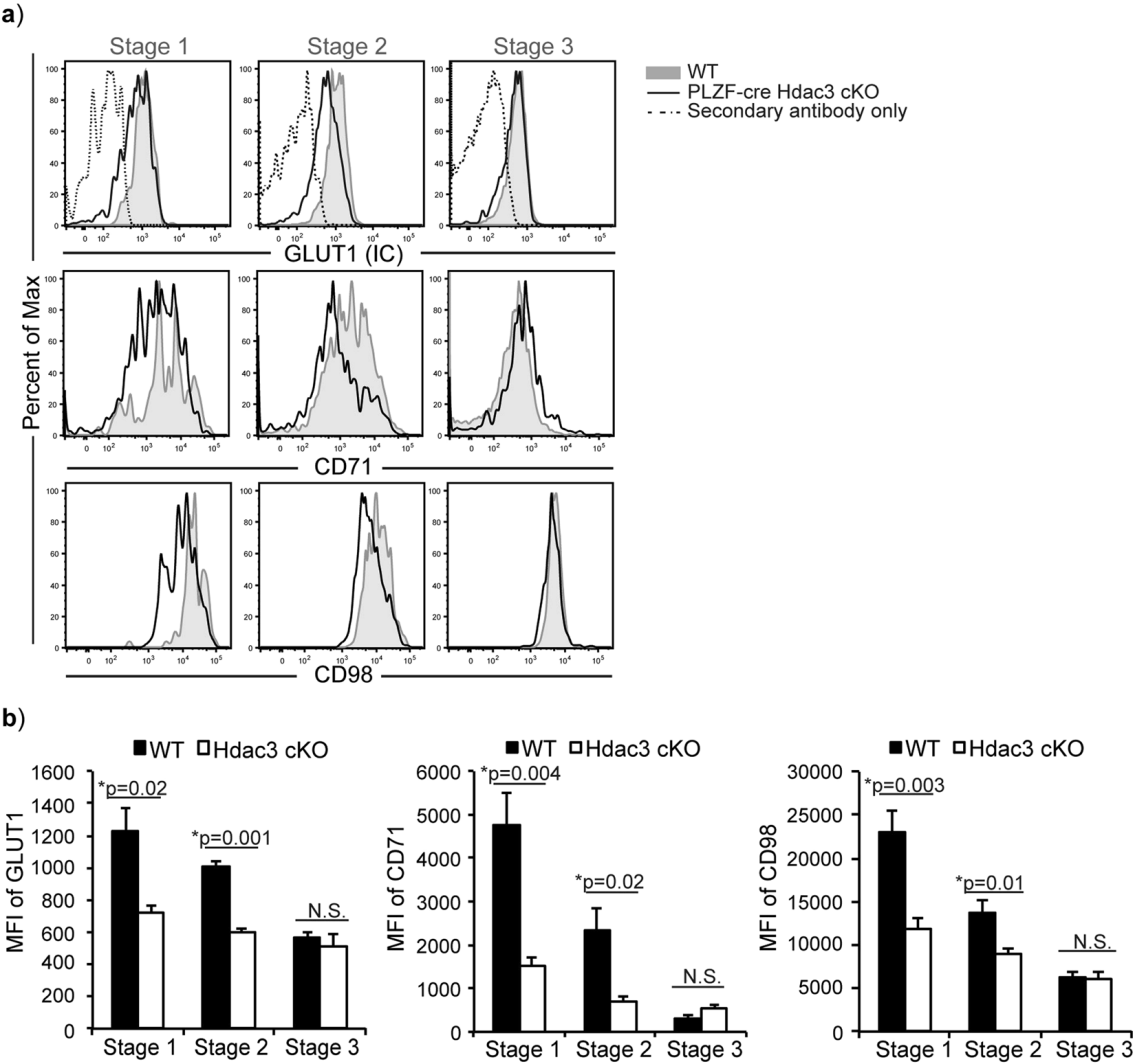
Loss of Hdac3 affects expression of nutrient receptors CD71, CD98 and GLUT1. (**a**) Expression of GLUT1 (intracellular), CD71 (surface), and CD98 (surface) in Stage 1–3 iNKT cells of WT (grey filled) and PLZF-cre Hdac3 cKO (black line). Secondary antibody (fluorescent labeled) only control (dashed) is shown for GLUT1 where unlabeled primary antibody against GLUT1 was used. CD71 and CD98 antibodies were directly conjugated to fluorophores. Data is representative of at least 4 mice/genotype from 3 independent experiments. Staining and gating for each iNKT cells stages was performed as described in Fig. 1. (**b**) Quantification of average MFI of GLUT1, CD71 and CD98 expression in Stage 1–3 iNKT cells of WT (black bars) and PLZF-cre Hdac3 cKO (white bars) mice. Data is calculated from 4 mice/genotype from 3 independent experiments. Statistical analysis was done using student’s *t* test. Means ± SEM.

Autophagy is an important cellular process required for cell homeostasis and can serve as an alternate source for energy production during nutrient deprivation. Thus, cells defective in autophagy could become hypersensitive to nutrient deprivation. In PLZF-cre Hdac3 cKO mice, there was reduced expression of nutrient receptors GLUT1, transferrin receptor (CD71) and large neutral amino acid transporter (CD98) in Hdac3-deficient iNKT cells as compared to WT (Fig. 6a). Quantification of MFI of GLUT1, CD71 and CD98 expression revealed significant reduction in Hdac3-deficient iNKT cells compared to WT (Fig. 6b). Altogether, the reduced expression of nutrient receptors suggests that Hdac3-deficient iNKT cell may be experiencing nutrient deprivation; and thus when coupled with reduced autophagy, could be the cause for the severe defect in iNKT cell development in the absence of Hdac3.

## Discussion

In this study, we demonstrate the essential role of Hdac3 in iNKT cell development. In PLZF-cre Hdac3 cKO mice there is a dramatic decrease in the proportion and absolute cell number of iNKT cells. In particular, there is an over 10-fold reduction in the number of NKT1/Stage 3 iNKT cells in the absence of Hdac3. The reduction in absolute cell number is not due to a defect in proliferation but instead is a result of increased cell death. The loss of NKT1/Stage 3 iNKT cells is also not due to dysregulated IL-15 mediated homeostasis or lower expression of Bcl-2, as neither a Bcl-xL transgene nor a Bcl-2 transgene rescued the defect in iNKT cell numbers in PLZF-cre Hdac3 cKO mice. Rather, Hdac3-deficient iNKT cells have reduced autophagy that may be exacerbated by decreased expression of nutrient receptors GLUT1, CD71 and CD98, and thus may have lead to decreased survival.

Conventional naïve T cells go through metabolic reprograming during antigen recognition leading to activation and differentiation into effector T cells^40, 41^. Upon activation they switch to aerobic glycolysis from oxidative phosphorylation to meet the increased energy demand during proliferation and effector differentiation. During the contraction phase when effector T cells differentiate into memory T subsets, they switch back to oxidative phosphorylation as the demand for energy decreases. During development, iNKT cells also undergo metabolic reprogramming and require autophagy for their transition to a quiescent state after population expansion^21, 23^. They require more energy during Stage 1 when they are proliferating while reducing metabolic demand as they acquire memory phenotype and differentiate into effector subsets. Stage 3 iNKT cells are long lived resident cells in the thymus, exhibiting a quiescent phenotype and thus rely on autophagy for survival. Salio *et al*. showed that proliferating Stage 1 iNKT cells required more glucose while maturing Stage 2 and Stage 3 iNKT cells reduced their glucose uptake and increased their autophagy levels^21^. Furthermore, mice with mutations in key autophagy genes have a severe block in iNKT cell development at Stage 2, supporting the requirement of autophagy at later developmental stages.

Autophagy is an evolutionarily conserved cellular process important for cell homeostasis. It is a process where intracellular components such as cytoplasmic materials are degraded and recycled to maintain cell survival, differentiation and growth^18^. Autophagy can be activated when cells undergo metabolic stress such as energy starvation to provide an alternate energy source. Hdac3-deficient iNKT cells have a reduction in autophagy, which could lead to increased requirement for nutrient uptake for their survival. The inability to increase their nutrient uptake during their need to correct the reduced autophagy may be exacerbating cell death observed in Hdac3-deficient iNKT cells. Loss of the requisite autophagy gene Atg7 in iNKT cells leads to increased cell death. The increased cell death in the Atg5-deficient iNKT cells was not rescued by the transgenic expression of Bcl-xL^23^. The severe decrease in iNKT cell numbers in PLZF-cre Hdac3 cKO mice was also not rescued by either a Bcl-2 or Bcl-xL transgene, similar to the Atg5-deficient iNKT cell defect. This further suggests that apoptosis induced by a reduction in autophagy involves more than the balance of the Bcl-2 family genes to promote survival. Although being essential for iNKT cell development, the regulation of autophagy in iNKT cells is undefined. It was suggested that modulation of iNKT cell metabolism by mTOR pathways and metabolic regulators, LKB1 and Fnip1 may play a role^42–45^. However, in those studies it was not established if there was a defect in autophagy in iNKT cells.

The loss of either Atg5 and Atg7 in iNKT cells lead to significant decrease in NKT1 cell numbers with little alteration to the NKT2 and NKT17 numbers which is similar to the PLZF-cre Hdac3 cKO mice. Moreover the loss of Atg7 also leads to a decrease in IL-4 and IFN-γ production, which is also similar to Hdac3-deficient iNKT cells with severe reduction in IL-4 and IL-17 production. Thus autophagy is important for iNKT cell function to produce appropriate amount of cytokines upon stimulation. Autophagy regulated by the gene *PIK3C3* (also known asVps34) is also important for iNKT cell development at the earliest Stage 0^22^. The block in iNKT cell development in CD4-cre Vps34 cKO mice was cell intrinsic and independent of CD1d dependent antigen presentation. Although Vps34 plays a major role in autophagy, it is also crucial for other cellular processes such as endocytic trafficking^46^. The loss of Vps34, early in T cell development, using Lck-cre, lead to a block in T cell development that was independent of its function in autophagy^47^. In that study, Vps34 was important for trafficking of the survival cytokine IL-7Rα in T cells. Because Vps34 has complex functions in cellular biology besides autophagy, it is possible that the role of Vps34 in early iNKT cell development is more complex than its role in autophagy alone. Thus, in early iNKT cell development the role of Vps34, in regards to its function in autophagy, may be distinct than the role of autophagy mediated by Atg5 and Atg7 as the loss of latter genes cause a block in later Stage 2 iNKT cell development. This further supports the intricate role of autophagy mediated by different genes to regulate early and late stages of iNKT cell development. Defects in autophagy in other cell types have lead to a defect in MHC II antigen presentation, however the loss of Hdac3, does not lead to decreased expression of CD1d molecule on iNKT cells of PLZF-cre Hdac3 cKO mice (Supplemental Fig. 1). Although the expression of Atg7 was unaltered in iNKT cells of PLZF-cre Hdac3 cKO mice, over 30 genes have been identified to regulate autophagy, thus the transcriptional regulator, Hdac3 may regulate other autophagy related genes yet to be defined for iNKT cell development^48^. Thus, we have defined a potential role for Hdac3 in regulating autophagy for iNKT cell development and survival.

Previously we showed that transcriptional repressor NKAP associates with Hdac3 for its repressor function and NKAP has multiple roles in the development and differentiation of iNKT cells^31, 49^. In CD4-cre NKAP cKO mice, there is a complete block in iNKT cell development while the development of conventional T cells to CD4 and CD8 SP thymocytes is normal. We demonstrated that NKAP is required for positive selection of DP thymocyte into the iNKT cell lineage. We showed that deletion of Hdac3 using CD4-cre model also leads to a similar defect in iNKT cell development, reminiscent of CD4-cre NKAP cKO mice. In CD4-cre Hdac3 cKO mice, there is a complete block in iNKT cell development while the development of conventional CD4 and CD8 SP thymocytes was normal. This suggested that NKAP and Hdac3 may work together to regulate positive selection of DP thymocytes into iNKT cell lineage. However, we now have demonstrated that after positive selection into the iNKT cell lineage, NKAP and Hdac3 work independently of each other in later iNKT cell development and differentiation. Deletion of NKAP using PLZF-cre leads to a block in iNKT cell proliferation and specific defect in the differentiation of ROR-γt expressing NKT17 cells. The phenotype in PLZF-cre NKAP cKO is different than what we observe in PLZF-cre Hdac3 cKO mice. Hdac3-deficient iNKT cells have normal proliferation and can differentiate into NKT17 cells. However, in the absence of Hdac3, there is a reduced autophagy, survival and cytokine production by NKT2 and NKT17 cells not observed in the absence of NKAP. Thus, although Hdac3 and NKAP may work together to mediate positive selection of iNKT cells, Hdac3 works independently from NKAP later in iNKT cell development. Hdac3 may associate with other transcriptional regulators and hence may regulate autophagy in iNKT cell for their development and survival.

## Methods

### Mice

The generation of PLZF-cre^33^, Hdac3^fl/fl^ ^50^, Rag1-GFP^51^, Bcl-2 Tg^52^, Bcl-xL Tg^53^ has been described. All mice were on C57Bl/6 genetic background. Mice were housed in barrier facilities and all experiments were performed in Mayo Clinic with the approval of the Institutional Animal Care and Use Committee. All mice were analyzed at 5–16 weeks of age, with controls being PLZF-cre or Hdac3 fl/fl littermates whenever possible, or age-matched Hdac3 fl/fl non-littermates.

### Ethics Approval

All animal experiments in this study were approved by the Institutional Animal Care and Use Committee of Mayo Clinic. All procedures were performed in accordance with approved guidelines and regulations from the Mayo Clinic.

### Flow Cytometry

All flow cytomtery were performed either on the Attune NxT (Life Technologies) or LSRII (BD Science) and analysed in FlowJo (Tree Star). Each analysis had doublet cells excluded and live/dead gating using fixable viabilty dye (Tonbo) before analysis, unless otherwise stated. Antibodies were bought either conjugated to fluorophore or unconjugated from Tonbo, BD Bioscience, eBioscience, Biolegend, Cell Signaling Technology, AbCam or R&D Systems. The following flow cytomtery antibodies were purchased as follows: Tonbo: TCR-β (H57.597), CD4 (RM-5, GK1.5), NK1.1 (PK136), IL-4 (11311), IL-17A (TC11-18H10-1), IFN-γ (XMG1.2). eBioscience: Ki-67 (SolAl5), CD132 (TUGm2), IL-15Rα (DNT15Ra), Egr2 (erongr2), CD98 (RL388), CD71 (RL7217), Annexin V, CD24 (M1/69), NK1.1 (PK136), PLZF (Mags.21F7), Tbet (4B10), Bcl-2 (10C4). Biolegend: CD44 (IM7), CD8α (53–6.7), CD1d (1B1), CD122 (TM-B1), CD24 (M1/69), IL-4 (11311), IL-17A (TC11-18H10-1), IFN-γ (XMG1.2), PLZF (Mags.21F7, 9E12), Tbet (4B10), Bcl-2 (10C4), CD4 (RM-5, GK1.5). Cell Signaling Technology: Egr1 (44d5), Mcl-1 (D2W9E), Bim (C34C5), LC3A/B (D3U4C), c-Myc (D84C12), Anti-Rabbit IgG (AF647). R&D Systems: NK1.1 (KLRG-1C), Atg7 (683906), Isotype (ATG7) IgG1 #1711 (MAB002). AbCam: Hdac3 (ab7030), Isotype (Hdac3) IgG (ab171870), Bcl-xL (7B2.5), p62 (ab56416), Isotype (p62) IgG2a mouse FITC (ab9136). BD bioscience ROR-γt (B2D). For experiments where unconjugated primary antibody with fluorescent 2^nd^ antibody was used (such as c-Myc, Egr1, GLUT1), 2^nd^ only control for WT was also examined and is shown as a negative control. PE or BV421 conjugated CD1d:PBS57 or CD1d:empty tetramers were generously provided by the NIH Tetramer Facility. Cells were stained for fixable viablity dye (FVD) in 1x PBS before staining for surface antigens in FACS buffer. For intracellular stains, cells were stained for FVD and surface antigens before being fixed and permeablized with Foxp3 fix:perm buffer kit (eBioscience or Tonbo) and then stained for intracellular antigens. The following flow cytomtery antibodies were purchased as follows.

### Isolation of iNKT cells

Thymocytes were stained with PE-conjugated CD1d:PBS57 tetramer, then iNKT cells were positively selected using anti-PE coated magnetic beads (Miltenyi Biotec) and separated using (LS) MACS separation column. Isolated cells were then stained with TCR-β, BV421-CD1d:PBS57 tetramer, CD24, CD44 and NK1.1 to distinguish iNKT cell developmental stages. Gated iNKT cells (CD1d:PBS57^+^ TCR-β^+^) were distinguished in stages using Stage 1 (CD24^−^ CD44^lo^ NK1.1^−^) Stage 2 (CD24^−^ CD44^hi^ NK1.1^−^) Stage 3 (CD24^−^ CD44^hi^ NK1.1^+^). Cells were sorted directly into lysis buffer from RNeasy kit (Qiagen). Sorts were performed with FACSAria (Becton Dickinson) by the Mayo Clinic Flow Cytometry Core Facility.

### Quantitative PCR

cDNA was generated and amplified using an Ovation PicoSL WTA V2 kit (NuGen) from mRNA (Qiagen RNase mini kit) isolated from sorted iNKT cells. Taqman *Hdac3* and *Gapdh* gene expression assays were purchased from Applied Biosystems. The Taqman assay *Hdac3* spanned a deleted exon (exon 7) in the *Hdac3* gene. Relative expression of *Hdac3* was calculated using 2^−ΔΔCT^ method^54^, q-PCR was performed in ABI RT-PCR StepOne Plus System (Applied Biosystems). *Hdac3* expression was calculated relative to WT Stage 3 (=1).

### Stimulation of iNKT cells

For cytokine production, isolated thymocytes were stimulated or left unstimulated for total of 6 hrs in complete culture media (RPMI) with 200 nM PMA and 1 μM Ionomycin. After an hour into stimulation BFA/Monensin (BD) was added to stop cytokine secretion. Cells were harvested 5 hours later and stained for FVD and surface antigens before fixation/permeablization to stain for cytokines and intracellular antigens.

### Apoptosis

Apoptotic iNKT cells were identified using FVD and Annexin V staining. Thymocytes were first stained for FVD in 1× PBS for 30 min on ice and then stained for surface markers for iNKT cells in FACS buffer for 30 min on ice. After surface staining, cells were washed and resuspended in 1x binding buffer then stained with Annexin V for 30 min on ice. Cells were washed and immediately analyzed by flow cytometry.

### Autophagy

Thymocytes were stained for FVD and iNKT cell surface markers before staining with Cyto-ID autophagy detection kit (Enzo Life Science) according to the manufacturer’s instructions.

### Statistical analysis

Two-tailed Student’s *t* test was used for statistical analysis with *p* value < 0.05 considered significant.

## Electronic supplementary material

Supplementary Info
