# Supplementary material for: Histone deacetylase 3 is required for iNKT cell development
Source: Sci Rep. 2017 Jul 19;7:5784. doi: 10.1038/s41598-017-06102-5 (PMC5517478; doi:10.1038/s41598-017-06102-5)
Supplement: Supplementary file 1 — Supplementary Info [file 41598_2017_6102_MOESM1_ESM.pdf]

Supplementary information:

Histone deacetylase 3 is required for iNKT cell development

Puspa Thapa,[1] Sinibaldo Romero Arocha,[1,3] Ji Young Chung,[1,3] Derek B. Sant'Angelo,[2] and Virginia Smith Shapiro[1,*]

[1]Department of Immunology, Mayo Clinic, 200 First Street SW, Rochester, MN 55905.

[2]Department of Pediatrics, Rutgers Robert Wood Johnson Medical School and The Children's Health Institute of New Jersey, 89 French Street, Room 4273, New Brunswick, NJ 08901.

[3] S.R.A. and J.Y.C. contributed equally.

[*]To whom correspondence should be addressed:
Virginia Smith Shapiro Ph.D.
200 1st Street SW
Rochester, MN 55905
Phone: 507-293-0615
Email: shapiro.virginia1@mayo.edu

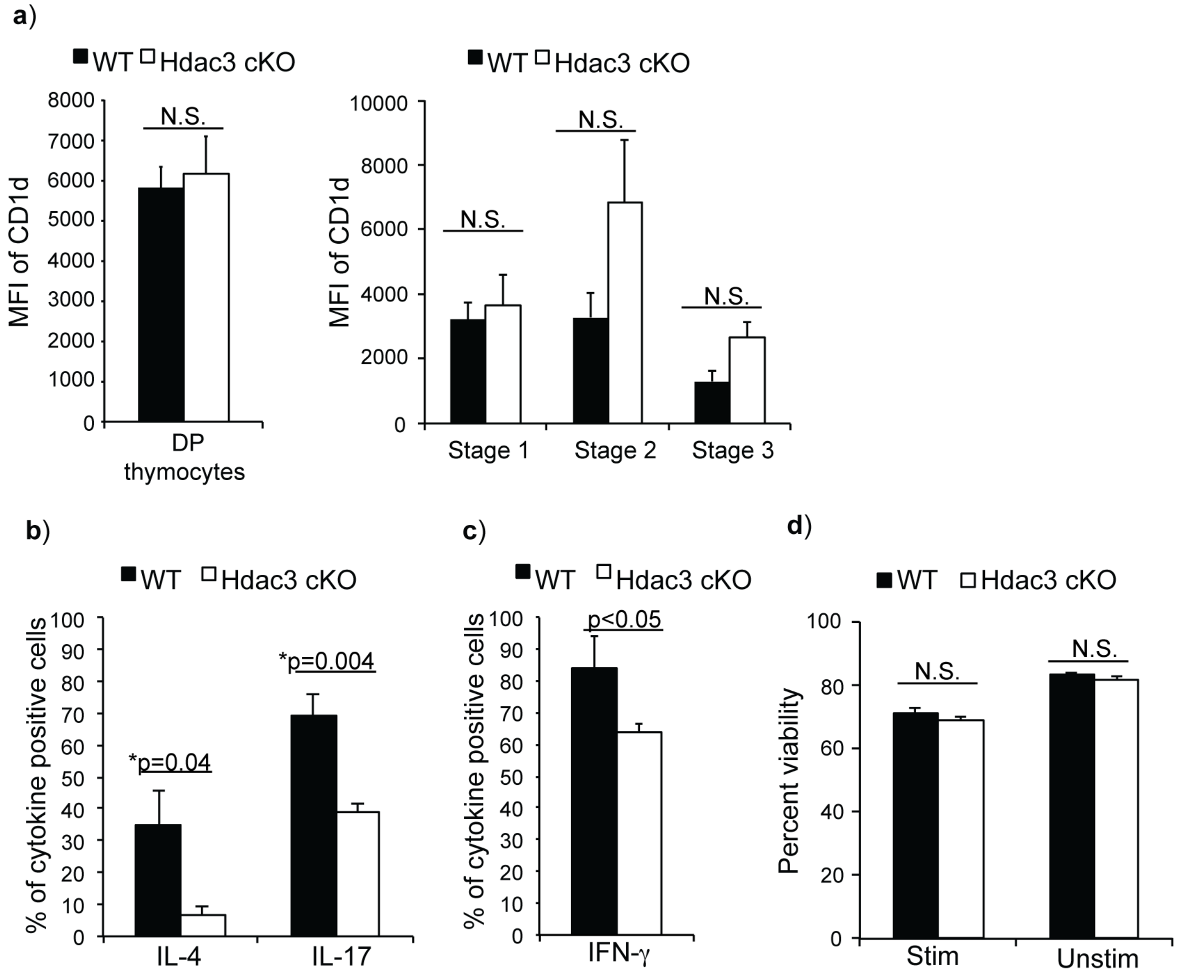

Supplemental Figure 1: Loss of Hdac3 does not lead to decreased expression of CD1d on DP thymocytes and iNKT cells. (**a**) Average MFI of CD1d expression on DP thymocytes of WT (black bar) and PLZF-cre Hdac3 cKO (white bar) mice. Data is calculated from 4 mice/genotype from 2 independent experiments**.** Statistical analysis was done using student's *t* test. Means ± SEM. (**b-c**) Quantification of proportion of cells positive for each cytokine (IL-4, IL-17 and IFN-γ). Data is calculated from 5 mice/genotype from 3 independent experiments. Statistical analysis was done using student's *t* test. Means ± SEM. (**d**) Quantification of viability of stimulated and unstimulated iNKT cells from WT (white bars) and PLZF-cre Hdac3 cKO (black bars) mice. Data is calculated from 5 mice/genotype from 3 independent experiments. Statistical analysis was done using student's *t* test. Means ± SEM.

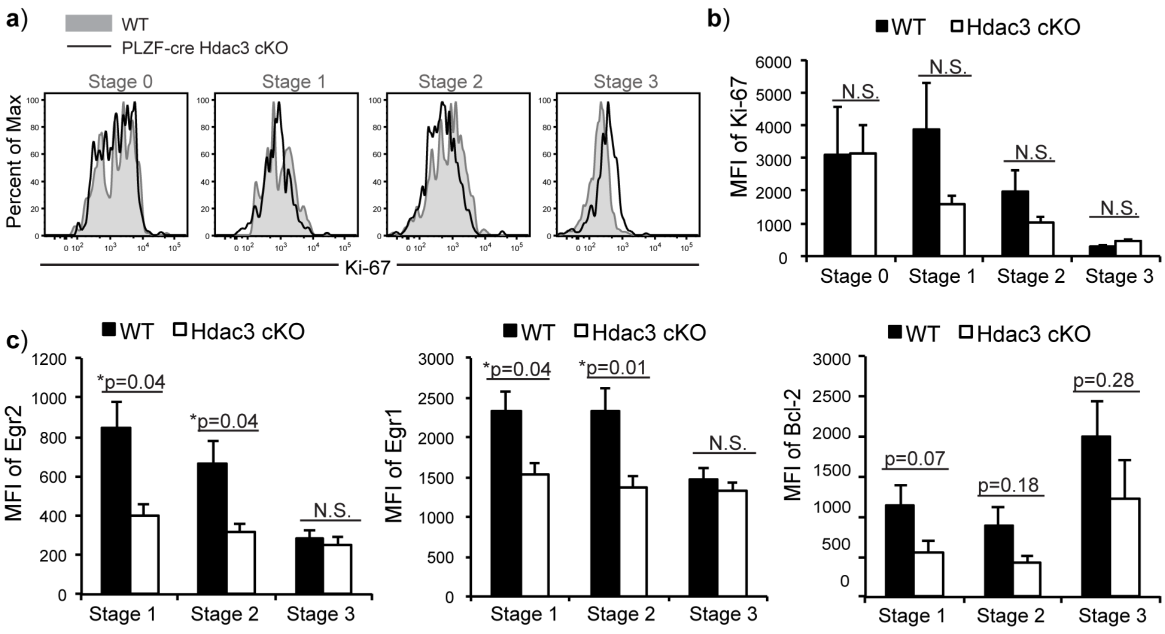

Supplemental Figure 2: Expression of Egr1 and Egr2 is significantly reduced in Hdac3-deficient iNKT cells. (**a**) Intracellular expression of Ki-67 in Stage 0-3 iNKT cells of WT (grey filled) and PLZF-cre Hdac3 cKO (black line) mice. Data is representative of 3 mice/genotype from 3 independent experiments. (**b**) Quantification of average MFI of Ki-67 in Stage 0-3 iNKT cells of WT (black bars) and PLZF-cre Hdac3 cKO (white bars) mice. Data is calculated from at least 3 mice/genotype from 3 independent experiments. Statistical analysis was done using student's *t* test. Means ± SEM. (**c**) Quantification of MFI of Egr2, Egr1 and Bcl-2 in Stage 1-3 iNKT cells of WT (black bars) and PLZF-cre Hdac3 cKO (white bars) mice. Data is calculated from at least 3 mice/genotype from 3 independent experiments. Statistical analysis was done using student's *t* test. Means ± SEM.

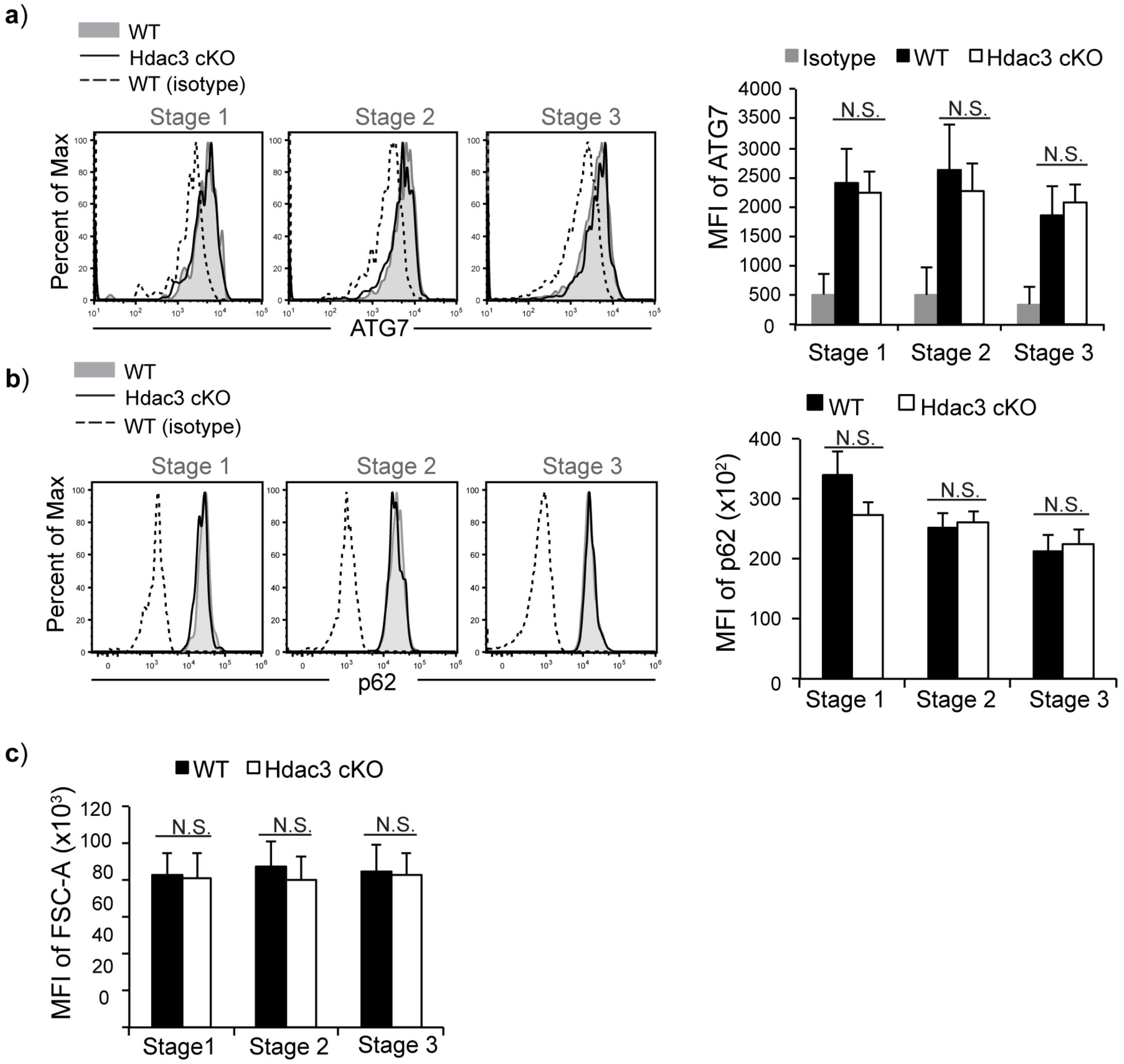

Supplemental Figure 3: Loss of Hdac3 does not alter the expression of ATG7 and p62 in Hdac3-deficient iNKT cells. (**a**) Expression of autophagy related gene ATG7 in Stage 1-3 iNKT cells in WT (grey filled) and PLZF-cre Hdac3 cKO (black line) mice. Unconjugated isotype control (dashed) for ATG7 is shown as a negative control. Secondary antibody (fluorescent labeled) was used to label both isotype and ATG7 primary antibodies. Data is representative of 4 mice/genotype from 3 independent experiments. Quantification of average MFI of ATG7 in Stage 1-3 iNKT cells of WT (black bars), PLZF-cre Hdac3 cKO (white bars) mice and WT, isotype control (grey filled bars). Data is calculated from at least 3

mice/genotype from 3 independent experiments. Statistical analysis was done using student's *t* test. Means ± SEM. (**b**) Expression of p62 in Stage 1-3 iNKT cells in WT (grey filled) and PLZF-cre Hdac3 cKO (black line) mice. Unconjugated isotype control (dashed) for p62 is shown as a negative control. Secondary antibody (fluorescent labeled) was used to label both isotype and p62 primary antibodies. Data is representative of 4 mice/genotype from 2 independent experiments. Quantification of average MFI of p62 in Stage 1-3 iNKT cells of WT (black bars) and PLZF-cre Hdac3 cKO (white bars) mice. Data is calculated from at least 4 mice/genotype from 2 independent experiments. Statistical analysis was done using student's *t* test. Means ± SEM. (**c**) Quantification of MFI of FSC-area in Stage 1-3 iNKT cells of WT (black bars) and PLZF-cre Hdac3 cKO (white bars) mice. Data is calculated from at least 3 mice/genotype from 3 independent experiments. Statistical analysis was done using student's *t* test. Means ± SEM.
